# Experimental dataset from a round robin test of contact parameters and hysteresis loops for nonlinear dynamic analysis

**DOI:** 10.1016/j.dib.2024.110374

**Published:** 2024-04-04

**Authors:** Alfredo Fantetti, Daniele Botto, Christoph Schwingshackl, Stefano Zucca

**Affiliations:** a Department of Mechanical Engineering, Imperial College London, Exhibition Rd, London, UK; b Department of Mechanical Engineering, Politecnico di Torino, Corso Duca degli Abruzzi, Torino, Italy

**Keywords:** Hysteresis loops, Friction coefficient, Contact stiffness, Reciprocating motion, Fretting, Nonlinear dynamics, Wear

## Abstract

This data article describes the extensive experimental dataset of friction hysteresis measured during the round robin test of the original research article [1]. The round robin test was performed on the two different fretting rigs of Imperial College London and Politecnico di Torino, and consisted of recording comparable friction hysteresis loops on specimen pairs manufactured from the same batch of raw stainless steel. The reciprocating motion of the specimens was performed at room temperature under a wide range of test conditions, including different normal loads, displacement amplitudes, nominal areas of contact and excitation frequencies of 100 Hz and 175 Hz. Friction forces and tangential relative displacements for each specimen pair were recorded and stored as hysteresis raw data. Each hysteresis loop was post-processed to extract friction coefficient, tangential contact stiffness and energy dissipated, whose evolution with wear was thus obtained and stored as well. MATLAB^Ⓡ^ scripts for post-processing and plotting data are included too.

The dataset can be used by researchers as a benchmark to validate theoretical models or numerical simulations of friction hysteresis models and wear mechanisms, and also to study the physics of friction hysteresis and its contact parameters. This friction data can also be used as input in models for nonlinear dynamics applications as well as to provide information on the contact measurement uncertainty under fretting motion. Other applications include using this data as a training set for machine learning applications or data-driven models, as well as supporting grant applications.

Specifications Table

**Table:** 

Subject	Mechanical Engineering / Surfaces and Interfaces
Specific subject area	Surface science and engineering concerning tribology, wear and friction hysteresis of dry metallic contacts
Data format	Raw data in .dat and .tdms files (dataset with numbers) Post-processed data in .mat files, .fig figures, .avi videos and power point
Type of data	Table, Image, Graph, Video, Figure
Data collection	Steel specimen pairs were tested under reciprocating motion from the two different fretting rigs built in-house in the Dynamics Group at Imperial College London and in the AERMEC Group at Politecnico di Torino. Laser doppler vibrometers were used to record the relative velocity of the specimens under reciprocating motion, and static and dynamic force transducers were used to record forces. The recorded friction hysteresis loops were post-processed with ad-hoc MATLAB^Ⓡ^ scripts to obtain 1) values of friction coefficients and tangential contact stiffness, and 2) the energy dissipated during the tests.
Data source location	Vibration University Technology Centre in Imperial College London, London, UK. AERMEC lab in Politecnico di Torino, Torino, Italy.
Data accessibility	Repository name: Dataset of contact parameters and hysteresis loops from a round robin test for nonlinear dynamic analysis Data identification number: 10.17632/gy587m7gx7.1 Direct URL to data: https://data.mendeley.com/datasets/gy587m7gx7/1
Related research article	Fantetti A., Botto D., Zucca S., Schwingshackl C., Guidelines to use input contact parameters for nonlinear dynamic analysis of jointed structures: Results of a round robin test, Tribology International, 2023. https://doi.org/10.1016/j.triboint.2023.109158

## 1 Value of the Data

• Measurements from this round robin test are valuable because there is a lack of direct comparisons of hysteresis data measured from fretting rigs at different institutions. The reliability of existing measurements is in fact partly limited by the individual capabilities of each specific test rig. These friction rigs do not often cover all measurement ranges of interest. In addition, a lack of direct comparisons between different rigs and a lack of a standardized approach for conducting measurements lead to low confidence in the measured parameters. Data from this round robin provides hence a unique comparison of measurements, taken from two different rigs on specimens manufactured from the same batch of stainless steel. The factors that may cause differences in the measurements from the different test rigs have been instead discussed in detail in the related research article [1].
• This data will particularly benefit researchers in the fields of joint mechanics, nonlinear dynamics, tribology and contact mechanics, who need to quantify contact parameters for their applications. For example, researchers working with structures subjected to vibration and made of several components in contact, such as turbomachinery, which require an accurate modelling of the contact behavior between vibrating contact interfaces.
• The dataset can be used as benchmark to validate theoretical models or numerical simulations of friction hysteresis models, contact parameters and wear evolution. The measured contact parameters can be used as input in contact models used for several applications, such as nonlinear dynamics of jointed structures.
• This data can be used to gain deeper insights into friction hysteresis, its contact parameters and their evolution in time. This understanding is critical for overcoming the lack of knowledge into the physics of friction contacts under vibration.
• The measurement results can serve as a benchmark for other research groups to participate in this comparative round robin study. More work is required to expand the test range of the round robin test and generalise the findings, also to other materials. By adding a further dataset, the statistical significance of the presented data can also be increased. Indeed, this data can be used to quantify the measurement uncertainty and increase the confidence in the measured contact parameters.
• The evolution of hysteresis loops with wear, for every loading condition, can be used as training set for machine learning applications or data-driven models, as well as supporting data for grant applications.

## 2 Background

Turbomachinery and other jointed structures are carefully designed to optimize their dynamic response and prevent unwanted high-cycle fatigue failures due to vibration. Advanced numerical models are employed to predict the often nonlinear dynamic responses, but their reliability is partially limited by the lack of understanding of the friction mechanisms between the vibrating contact interfaces. Although several fretting rigs have been developed at different institutions to measure contact parameters such as friction coefficient and contact stiffness, a lack of direct comparisons prevents a throughout understanding. To address this issue, a comparison of these contact parameters has been performed in [1] by employing the fretting rigs of Imperial College London and Politecnico di Torino. This data article adds value to the original research article [1] by making the raw and processed data publicly available [2] and reusable for further research and development.

## 3 Data Description

The data presented in this article is related to the friction hysteresis behavior of 80 stainless steel specimen pairs tested within a round robin test performed on the two different fretting rigs at Imperial College London [3] and Politecnico di Torino [4], hereafter referred to as Imperial and PoliTO respectively. Millions of hysteresis loops were recorded for each specimen pair at room temperature under an excitation frequency of 100 Hz for the Imperial rig and 175 Hz for the PoliTO rig. Each specimen pair was tested under a specific combination of the following loading conditions: four different normal loads (17 N, 87 N, 150 N and 253 N), four displacement amplitudes (1 µm, 14 µm, 25 µm and 50 µm) and four nominal areas of contact (1 mm^2^, 5 mm^2^, 10 mm^2^ and 40 mm^2^).

**Fig. 1:**
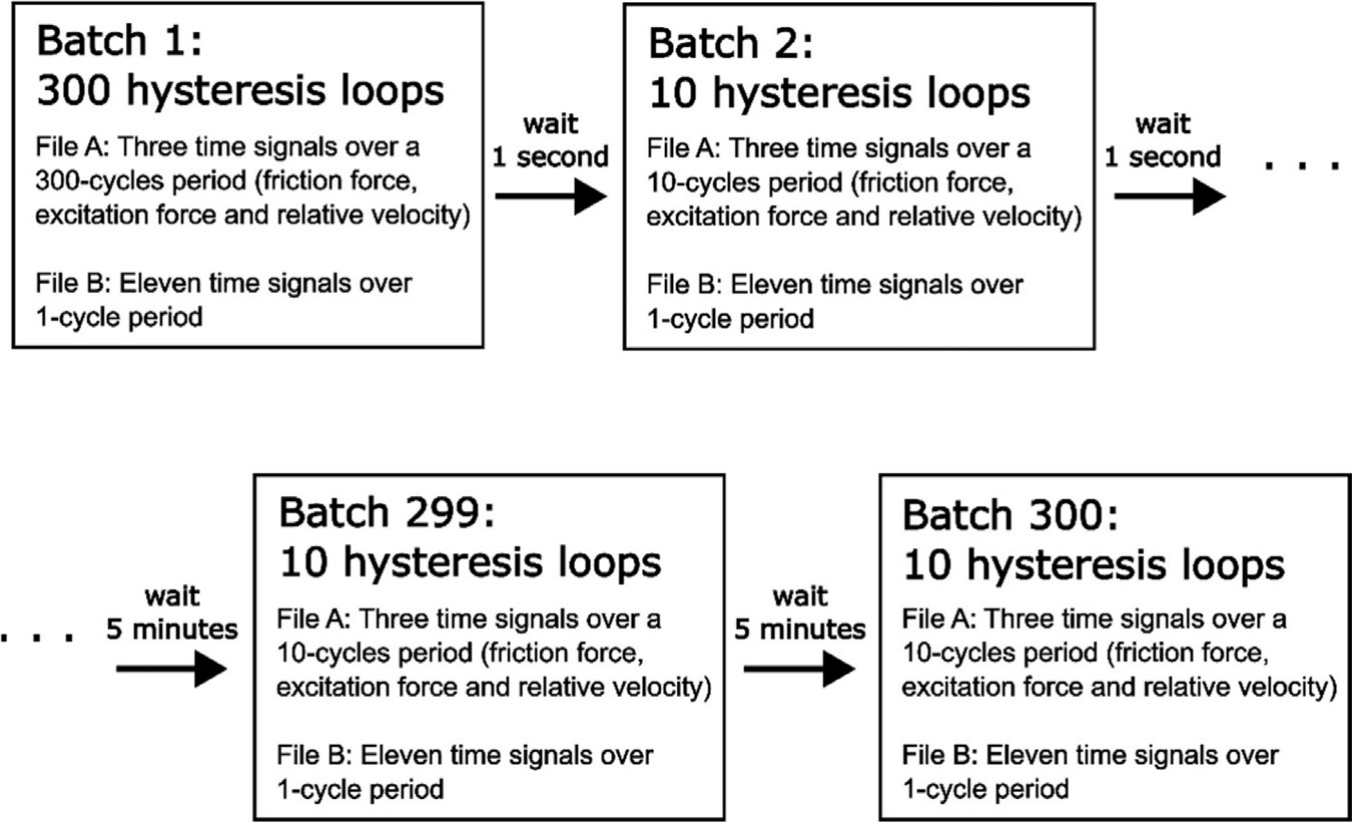
Recorded batches of hysteresis loops for each tested specimen pair. Within each batch, two file types were saved for tests performed at Imperial and one file type (not shown in the Figure) saved for tests performed at PoliTO.

Each of the 80 specimen pairs was tested for 2.5 consecutive hours (excluding additional repeatability tests performed on each pair). As a result, the completion of all the tests resulted in more than 200 h of testing that, at an average excitation frequency of 140 Hz, corresponds to roughly 100 million recorded hysteresis loops. Since recording all this data was unfeasible because of storage limits, loops were recorded once every 5 min in small batches of 10 consecutive loops. Only during the first 5 s of each test, all loops were continuously recorded. This was done because hysteresis loops strongly vary at the beginning of the test and consequently a high recording rate is needed to accurately capture their evolution [5]. After the first 5 s, loops were recorded with a lower rate until the 50^th^ minute, after which batches were recorded every 5 min, as shown in Fig. 1. This procedure is reasonably chosen because, after a running-in, a steady state is slowly reached [5]. At the end of the test, roughly 300 batches are recorded with this procedure.

Data from each test is stored in the “Imperial College London” and “Politecnico di Torino” folders. Each institution folder contains three subfolders: “raw_data”, which includes subfolders for every test with their respective recorded batches; “Post_processed_hysteresis”, which includes post-processed data such as the values of friction coefficients and contact stiffness extracted from the measured hysteresis loops; “Matlab scripts”, which contains MATLAB^Ⓡ^ scripts to post process and plot data. Both Imperial and PoliTO folders also contain one summary power point named “Imperial_SUMMARY.pptx” and “PoliTO_SUMMARY.pptx” respectively. These power points, described in this article, contain the numbering of each test in order to easily locate test numbers and plot them as desired.

The most important folder is the folder “SUMMARY” in “Post_processed_hysteresis”, which contains steady state data of the contact parameters and figures very helpful to have a clear snapshot of every tested specimen pair. These figures are also included in the two power points mentioned above. The Imperial and PoliTO folders only have few differences in the raw data format due to the different acquisition systems of the respective rigs. A detailed description of the Imperial folder is given below while, for the PoliTO folder, the few differences are described at the end of this section.

**Fig. 2:**
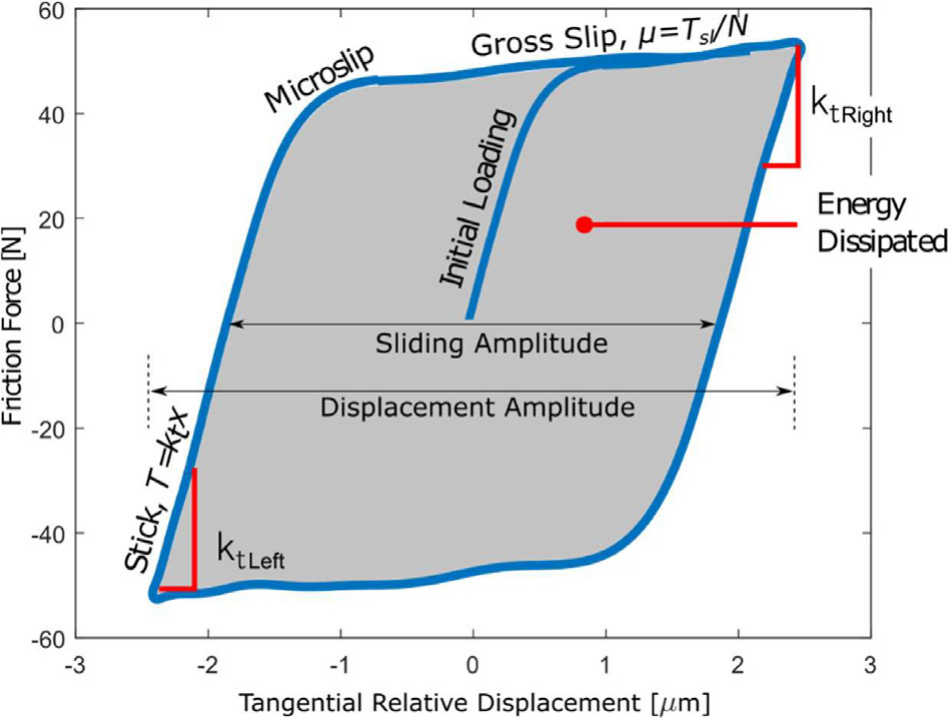
A typical hysteresis loop. *µ* is the friction coefficient, *T_sl_* is the friction force during gross slip, *N* is the normal load and *k_t_* is the tangential contact stiffness.
  ∘ *ExcitationFrequency* is the excitation frequency from the shaker (100 Hz in Imperial tests);
  ∘ *NormalLoad* is the normal load applied to the contact (85.5 N in this example);
  ∘ *ExcitationAmplitude* is the amplitude of excitation coming from the shaker (47.4 N in this example, at an excitation frequency of 100 Hz);
  ∘ *CumulativeEnergy* is the cumulative energy dissipated until that moment in the test (202.27 J in this example). The energy dissipated is the area inside the loop, as in Fig. 2. The cumulative energy is the sum of the area of each loop since the start of the test;
  ∘ *CycleNumber* is the number of the recorded cycle (180500^th^ hysteresis cycle in this example, which means roughly 30 min of running time for a 100 Hz excitation).
  Each *File A* type contains three columns with the time signals needed to plot several consecutive hysteresis loops. The first column is the measured friction force; the second column is the excitation force (although it is not needed to plot the hysteresis loops); the third column is the relative velocity between the two specimen pairs. By integrating the relative velocity, the relative displacement is obtained. By plotting the friction force versus the relative displacement, the hysteresis loop is obtained, as that shown in Fig. 2. In the case of Imperial, each hysteresis loop is composed of 600 datapoints since the sampling frequency of the recording instrumentation was 60 kHz and the excitation frequency was 100 Hz. Therefore, if 10 consecutive hysteresis loops are saved in this file, there would be three columns with 6000 values (600 datapoints for each of the 10 loops). During a typical test, roughly 300 batches are recorded with this procedure, resulting in millions of saved datapoints.
  This large amount of data has been read and post-processed with the MATLAB^Ⓡ^ script “read_raw_data_fileA.m” in the “Matlab scripts” folder. This script loads all the *File A* .dat files for a given tested specimen pair and plots the hysteresis loops by integrating the relative velocity of the specimen pairs to obtain their relative displacement. It is thus possible to plot the evolution of the hysteresis loops during the 2.5-hour test.
  Then, the script post-processes all the hysteresis loops and automatically extracts the contact parameters (friction coefficient and tangential contact stiffness). The contact parameter extraction is performed with the custom MATLAB^Ⓡ^ function “func_extract_contact_parameters.m” in the folder “Matlab scripts\functions”. Friction coefficient and tangential contact stiffness values have been obtained for every hysteresis loop of each analyzed test and have been saved in the folder “Post_processed_hysteresis,” described in the following section. The functioning of the MATLAB^Ⓡ^ scripts is instead described in the section “Data post processing with MATLAB^Ⓡ^ scripts”.
• *File B*: this second file type contains only one hysteresis loop per batch, but with eleven columns containing the time signals of all possible data recorded from the rig during the acquisition. The *File B* name has the following structure: “304_304_R1_25C_1mm2_14mum_100Hz_85.5N_xA_xB_Nbot_Nup_Fex_Tup_Tbot_vA_vB_aA_aB_180500cycle.dat”. In the file name there is the same information described for the *File A* type, plus the headers of the 11 columns contained in the file. The 11 columns correspond to the time signals of: *xA*, displacement of the top specimen; *xB,* displacement of the bottom specimen; *Nbot*, dynamic component of the normal load measured on the load cell placed below the bottom specimen; *Ntop*, dynamic component of the normal load measured on the load cell placed above the top specimen; *Fex*, excitation force from the shaker; *Tup,* friction force measured on the two load cells on the upper part of the static arm; *Fbot*, friction force measured on the load cell on the bottom part of the static arm; *vA, vB, aA* and *aB* are respectively the velocities of top and bottom specimens, and accelerations of bot and top specimens. In this case, each column contains the time signal of one full cycle, and therefore it is composed of 600 datapoints.

**Folder “raw_data” in “Imperial College London/”:** This folder contains one subfolder for each tested specimen pair numbered consecutively with prefix IC (e.g. “IC1”, “IC2” etc.). In each test folder, there are the batches recorded during the test. For each batch, two file types were created at the same time by the acquisition system: *File A* and *File B* as named in Fig. 1. Both files are in .dat format (binary text files) and contain measured displacements, friction forces and other measured data recorded continuously until the end of the test. The data content of the two file types is described as follow:

• *File A*: this file type contains the 10 (or more) consecutive hysteresis loops of the recorded batch. The File A name has a format like this: “304_304_R2_25C_1mm2_14mum_100Hz_85.5N_47.4Fex_202.27J_180500cycle.dat” and contains the information about the loading conditions. This naming corresponds to the following structure *Material1_Material2_Repeat_Temperature_NominalArea_Displacement_ExcitationFrequency_NormalLoad_ExcitationAmplitude_CumulativeEnergy_CycleNumber*. Where:
  ∘ *Material1* and *Material2* are the materials of the top (mobile) and bottom (fixed) specimen in the rig (always 304 stainless steel for every test);
  ∘ *Repeat* is the repeat number of the test since the same specimen pair was tested up to 5 times, by disassembling and reassembling it in the rig under the same loading conditions for repeatability analysis (in this example the test repeat is the number two);
  ∘ *Temperature* is the temperature at which the test was performed (25 C° for every test);
  ∘ *NominalArea* is the size of the nominal area of contact of the specimen pair (1 mm^2^ in this example);
  ∘ *Displacement* is the displacement amplitude of the hysteresis loops (14 µm in this example). The displacement amplitude is defined as in Fig. 2;

The content of these .dat files can be read with the MATLAB^Ⓡ^ script “read_raw_data_fileB.m” in the “Matlab scripts” folder. This script loads all the *File B* .dat files for a given tested specimen pair and plots the time signals of the different recorded variables. More details about this MATLAB^Ⓡ^ script are given at the end of this section.

• In addition to the .dat files, in some test folders there is a “log.txt” file in which issues that occurred during that specific test are reported, if any occurred.

**Folder “Post_processed_hysteresis” in “Imperial College London/”:** This folder contains one subfolder per test with the post-processed data for that test. Test folders are numbered consecutively with prefix IC (e.g. “IC1”, “IC2” etc.). Each test folder contains:

• One single file named “cycle.mat” made of two rows and several columns. The first row indicates the cycle numbers of the last hysteresis loop recorded for every batch. The second row contains the cumulative energy dissipated until that batch. For example, the folder “post_processed_hysteresis/IC1” contains one file “cycle.mat” with two rows and 313 columns indicating cycle number and cumulative energy dissipated of the 313 recordings during the 3 h of the test.
• One single file named “titolo.mat” with the name of the first batch saved for that test in the format “304_304_R2_25C_1mm2_14mum_100Hz_85.5N_47.4Fex_0.27J_300cycle”. This is needed to know information about the test in case the “raw_data” folder is not available.
• Several files with all the post processed information of each recorded batch. The name of these files is “cycle300.mat”, “cycle350.mat” etc. where the number indicates the cycle number of the last hysteresis loop recorded for that batch (which is also indicated in the first row of the file “cycle.mat”). This .mat files contain 4 variables:
• “hyst” is a matrix 600×2 containing the relative displacement (first column) and friction force (second column) needed to plot the last measured hysteresis loop of that batch. For example, for the test “IC1” in the “raw_data” folder, there are 10 recorded hysteresis loops for the recording “304_304_R1_25C_1mm2_14mum_100Hz_87.5N_47.5Fex_0.14J_350cycle.dat”. Therefore, the file “cycle350.mat” in the “post_processed_hysteresis” folder contains a 600×2 matrix in the “hyst” variable that plots the 10^th^ hysteresis loop for that recording. By plotting the loop of the “hyst” variable in each .mat file in that test folder, it is possible to see the evolution of the hysteresis loops in time for that test.
  ∘ “corners” is a vector of four numbers referring to the indexes used to extract the contact parameters for the hysteresis loop saved in “hyst” (see the section “Data post-processing with MATLAB^Ⓡ^ scripts”, which shows how hysteresis loops where post-processed through those four points to extract the contact parameters).
  ∘ “mu_Fs_ktL_ktR_m1_Fex_slip_muE” is a matrix n by 8, with n rows (one row for every post processed hysteresis loop in that recorded batch). The 8 columns contain the contact parameter values for each loop ordered as indicated in the variable name: *mu* is the friction coefficient extracted with the standard method (see the section “Data post-processing with MATLAB^Ⓡ^ scripts”), *ktL* and *ktR* are the left and right tangential contact stiffness, *m1* is the slope of the macroslip region, *Fex* is the excitation amplitude, *slip* is the displacement amplitude, *muE* is the friction coefficient extracted with the energy method. These values can be plotted for each .mat file to show the evolution in time of those parameters for every test.
  ∘ The values in this matrix have been analysed with the MATLAB^Ⓡ^ script “get_steadystate_values.m”, which extracted the steady state values of friction coefficient and tangential contact stiffness and saved them in the subfolder “SUMMARY” for further analyses.
  ∘ “k_Dx_T_dV_INSTANTS” includes a 3D matrix of size 599 by 4 by n, where n is the number of post processed hysteresis loops in that batch, 599 are the datapoints of those loops and the 4 columns in each matrix indicate respectively the instantaneous contact stiffness *k* (i.e. the slope between two consecutive force-displacement points), the relative displacement time signal *Dx*, the friction force time signal *T* and the relative velocity *dV. Dx* and *T* are needed to plot all the post processed hysteresis loops (of which the last of them is the same shown in the variable “hyst”).

The folder “Post_processed_hysteresis” also contains:

• An excel file named “Wear_analysis” that contains information on the worn area of contact of each specimen pair used in the experiments. The columns are: *Test*, with the test number; *Nominal area* is the nominal interface area calculated with the optical scans performed before the experiments; *Worn area Mob [mm2]* and *Worn area Fix [mm2]* are the worn areas of mobile (top) and fixed (bottom) specimens, estimated from the optical scans at the end of the experiments. The columns *Worn Volume Mob* and *Worn Volume Fix* do not contain values since the wear volume analysis has not been performed yet.
• A folder named “SUMMARY”, whose content is described as follow.

**Fig. 3:**
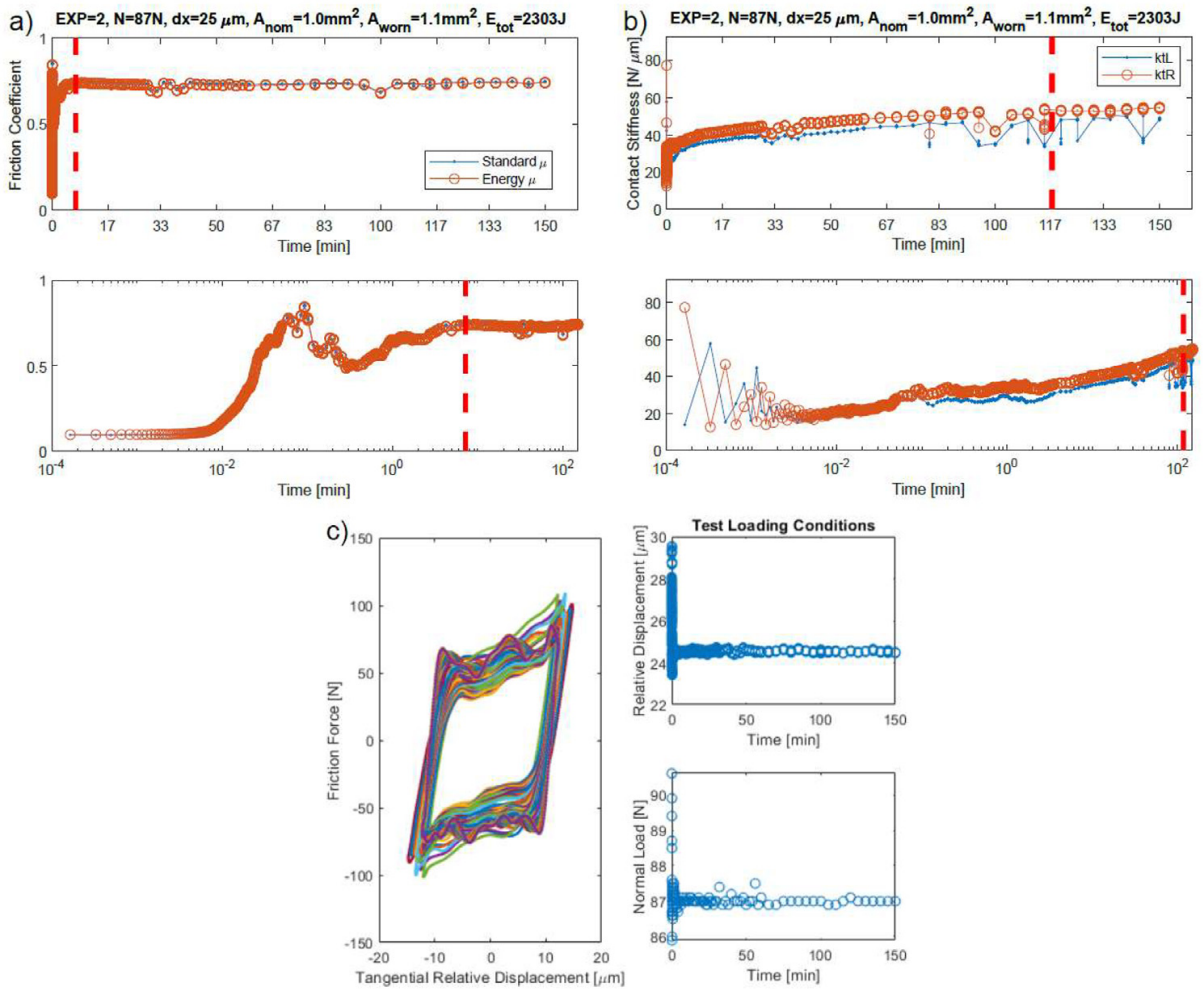
Examples of: a) mu.fig; b) kt.fig; c) loading_cond.fig. Figures for the test IC1.
    ▪ The .avi file is a video showing the evolution in time of each test. The name starts with the test number. For example, for the test number 1, the file name is “1evolution.avi”. The video shows the evolution in time of the hysteresis loops for a given test and the related friction coefficient and tangential contact stiffness. The video also shows if the loading conditions (normal load and displacement amplitude) changed during the test.

**Folder “SUMMARY” in “Imperial College London/Post_processed_hysteresis/”:** This folder is the most important folder and contains summary data for each test needed to study the round robin test results. For each test, there are one .mat file, one .avi file (video animation) and three .fig files (figures):

• The .mat file is named with the test number (“1.mat”, “2.mat” etc.) and contains three variables:
  ∘ “hyst” which is the last measured hysteresis loop for that test, which should correspond to a steady state hysteresis loop if a steady state was reached in that test.
  ∘ “input” is a table with the test information: normal load, displacement amplitude, running time, total energy dissipated, nominal area of contact, worn area of contact.
  ∘ “output” is a structure with one table and three vectors:
    ▪ The table is named “output.V_A_EkL_EkR_Emu” and shows the worn volume for that test (not analysed yet, so it is zero for every test), the worn area (obtained from the optical scans of the specimen interfaces at the end of the tests), and the energy needed to reach the steady state of left and right contact stiffnesses, and of the friction coefficient.
    ▪ The three vectors are named “kL”, “kR” and “mu” and contain the steady state values of contact stiffness left, contact stiffness right and friction coefficient respectively, measured after a steady state was reached. These values have been obtained with the MATLAB^Ⓡ^ script “get_steadystate_values.m” and are needed to run statistical analyses on the uncertainty of the measured contact parameters. These files are the most important and have been used to show all the trends in the friction coefficient and tangential contact stiffness in the original research article [1]. It is possible to study and plot these data with the two MATLAB^Ⓡ^ scripts “plot_SUMMARY_boxplot_figs.m” and “plot_SUMMARY_boxplot_figs.m”, as better described at the end of this section.
    ▪ The three .fig files are figures summarizing the test. Their name starts with the test number. For example, for the test number 1, the three names are “1mu.fig”, “1kt.fig” and “1loading_cond.fig”. The “mu.fig” and “kt.fig” show the evolution during the test of *µ* and *k_t_* respectively (see for example Fig. 3a,b), both in linear and log scale. Note that they have a vertical dashed red line. That line indicates the time point after which values for that contact parameter have been considered as steady state values (see the previous bullet point on the .mat file containing the steady state values). The “loading_cond.fig” shows all the hysteresis loops measured during the test and also the loading conditions in time, which should be constant over the whole test unless issues occurred during the test. See one example in Fig. 3c. These three Figures have been obtained for every test with the MATLAB^Ⓡ^ script “get_steadystate_values.m”.

**Power point “Imperial_SUMMARY.pptx” in “Imperial College London/”:** This power point is structured as follow:

• Slides 3–6: Photos of the Imperial friction rig and its specimens.
• Slides 7–9: Loading conditions of the round robin test.
• Slides 10–20: Overview of the results. Three slides for each nominal area of contact, of which the first and second slides show the test number for each loading combination, the end-of-test interface scans, the typical hysteresis loops and comments on the tests if present (see e.g. slide 11–12 for the 1 mm^2^ nominal area tests); the third slide contains the steady state values of contact stiffness (L: left, R: right) and friction coefficient (energy and standard method calculation), and also the worn area of contact (see e.g. slide 13 for the 1 mm^2^ nominal area tests). Tests performed at 1 µm do not have a friction coefficient or worn area of contact since they were fully stuck.
• Slides 21–43: Normal load comparison. These slides compare tests with the same nominal area of contact and same displacement amplitude but different normal loads. For each test combination, there are two slides: the first slide shows boxplots of the steady state values of the contact parameters and the second shows the evolution in time of the contact parameters. For example:
  ○ The slide 35 (first slide for a given test combination) compares tests performed under 24.5 µm displacement amplitude on specimen pairs with a 5 mm^2^ nominal contact area, but different normal loads. Plots in that slide show the boxplots of the steady state values of the friction coefficient and tangential contact stiffness (measured on both left and right part of the hysteresis loops). The plots on the right show the energy required to reach the steady state for each of the contact parameters in each test. These plots are obtained with the MATLAB^Ⓡ^ script “plot_SUMMARY_boxplot_figs.m” described at the end of this section.
  ○ The slide after (36 in this case) is always related to the previous and shows the evolution in time of the contact parameters for each test. That slide is needed to understand why some steady state values have certain behaviors. For example, in the slide 35, the contact stiffness measured at 17 N (both left and right) has many outliers. In the slide 36, the test n. 33 (which is the test performed at 17 N) shows a large amount of noise in both friction coefficient and contact stiffness because those tests experienced chattering, which led to very noisy hysteresis loops. The figures in slide 36 also show with vertical dashed red lines the time point from which a steady state has been reached for that contact parameter (the steady state has been chosen manually during post-processing as it will be explained in the following section). These figures are the same saved in the “SUMMARY” folder for each test in the format “1kt.fig” and “1mu.fig” as described above, which have been obtained with the MATLAB^Ⓡ^ script “get_steadystate_values.m” described in the section “Post-processing with MATLAB^Ⓡ^ scripts”.
• Slides 44–66: Displacement amplitude comparison. This set of slides shows the same plots as above, but comparing tests with the same normal load and nominal areas of contact, and different displacement amplitudes.

**Folder “Matlab scripts” in “Imperial College London/”:** This folder contains five runnable MATLAB^Ⓡ^ scripts and one subfolder named “functions” with other MATLAB^Ⓡ^ scripts used as functions within the main scripts. The five main scripts are:

• Two scripts to post-process data: “postprocess_raw_data_fileA.m” and “get_steadystate_values.m”, which have been used to post-process the data and are described in detail in the section “Post processing with MATLAB^Ⓡ^ scripts”. There is no need to use these scripts unless the reader wants to study the post-processing in detail.
• Three scripts to plot data: “plot_raw_data_fileB.m”, “plot_SUMMARY_boxplot_figs.m” and “plot_SUMMARY_violin_figs.m”, which can be used to plot the data and are described as follow.

**Fig. 4:**
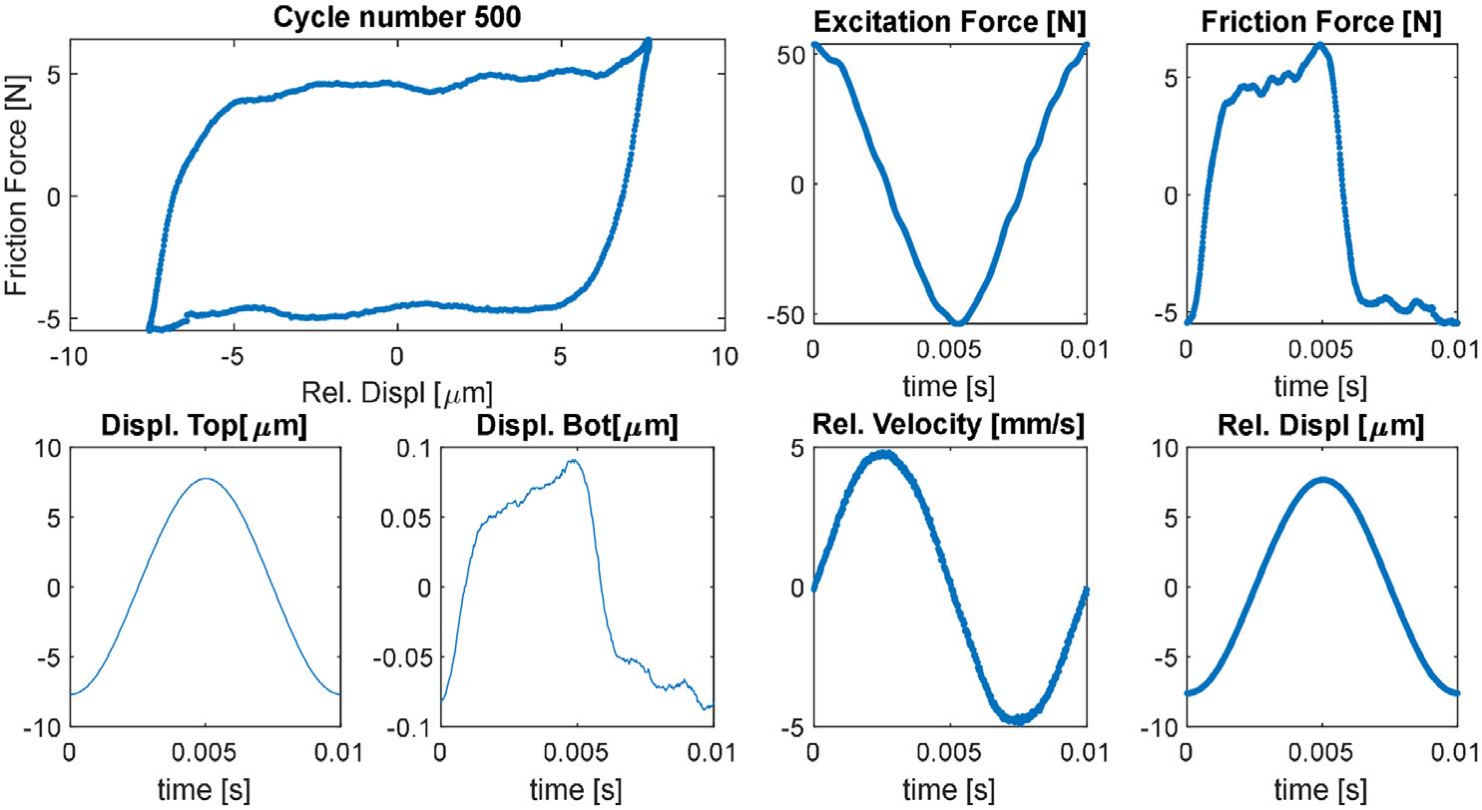
Variables plotted with the script “plot_raw_data_fileB.m”.

*Script “plot_raw_data_fileB.m”:* This script loads all the *File B* .dat files for a given tested specimen pair and plots the time signals of saved variables for each recorded batch. One figure for each recorded batch is plotted within a “for” cycle. By default, the variables plotted are those in Fig. 4. It is possible to plot additional variables by adding more figures in the “for” cycle. The user has only to choose the test and the variables to plot, which are described within the code itself. The description of the content of the *File B* type was provided in the description of the “raw_data” folder above.

**Fig. 5:**
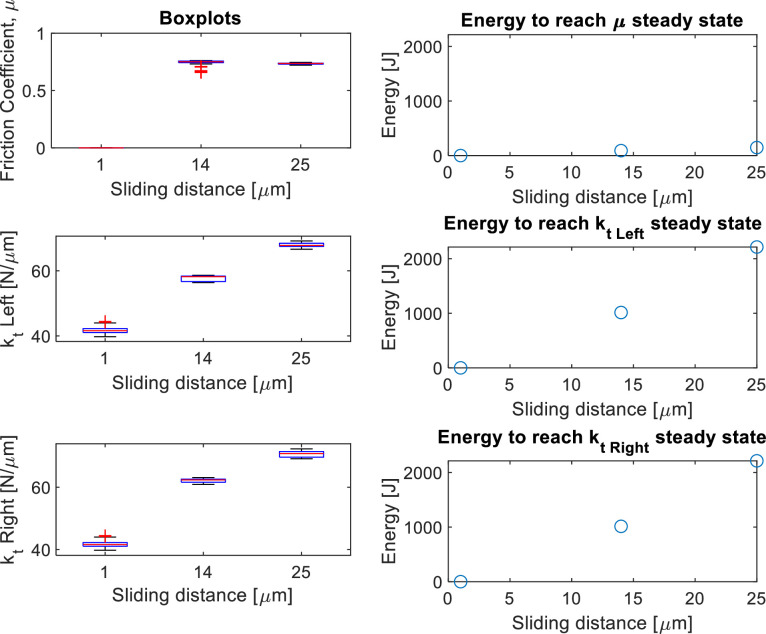
Boxplots versus displacement amplitude obtained with the script “plot_SUMMARY_boxplot_figs.m” for the test combination IC30-IC25-IC31. Each boxplot in the plots on the left corresponds to a different test and represents the steady state values of contact parameters versus the displacement amplitude. The plots on the right show the energy dissipated to reach the steady state in each test for the contact parameters.

*Script “plot_SUMMARY_boxplot_figs.m”:* This script plots the steady state values of friction coefficient and tangential contact stiffness for a chosen combination of tests. The steady state values are represented by means of one boxplot for each test plotted against the different loading conditions (namely normal load, displacement amplitude and nominal areas of contact). This script has been used to plot the boxplots in the “Imperial_SUMMARY.pptx” power point (slides 21–66) described above. An example of plots obtained with this code is shown in Fig. 5, which compares the tests IC30, IC25 and IC31 performed under 87 N normal load and 1, 14 and 24.5 µm displacement amplitudes respectively, on specimen pairs with a 5 mm^2^ nominal contact area. The three subplots on the left show the steady state values of friction coefficient and tangential contact stiffness measured on the left and right portions of the hysteresis loops respectrively. In each subplot, there is one boxplot per test, as indicated by the different displacement amplitudes of each test on the x-axis. The plots on the right show the energy required to reach the steady state for each of the contact parameters in each test.

**Fig. 6:**
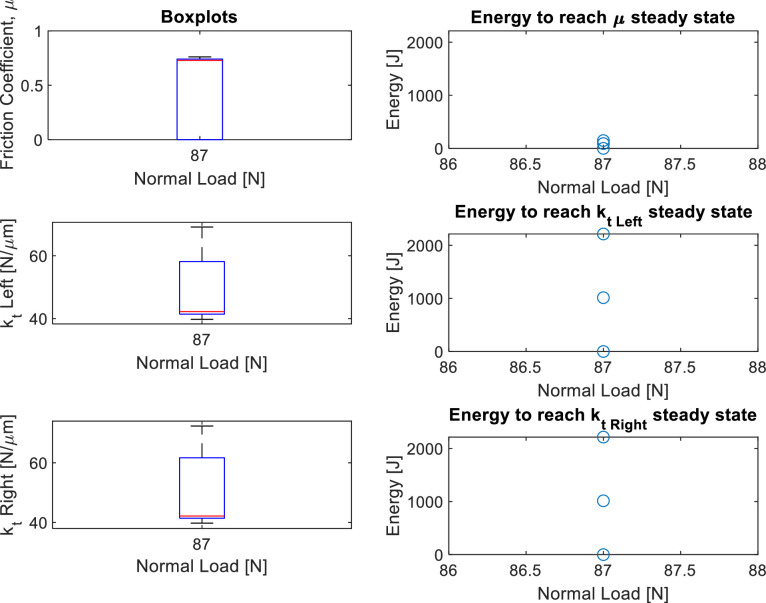
Boxplots versus normal load obtained with the script “plot_SUMMARY_boxplot_figs.m” for the test combination IC30-IC25-IC31. On the left, there is only one boxplot for the steady state values of the contact parameters. The boxplot includes the three tests since they were all performed at 87 N normal load. The plots on the right show the energy dissipated to reach the steady state in each test for the contact parameters.

The script generates these plots also using, for the x-axis, the normal loads and nominal and worn areas of contact of the tests. As an example, Fig. 6 shows the plots versus the normal loads. Since those tests were all performed at 87 N normal load, there is a single box plot including the steady state values of all three tests together.

The user can change the experiments to plot in the input of the script. The experiment numbers are summarized in the “Imperial_SUMMARY.pptx” power point (slides 11, 14 and 17), where the user can think of different test combinations to plot.

**Fig. 7:**
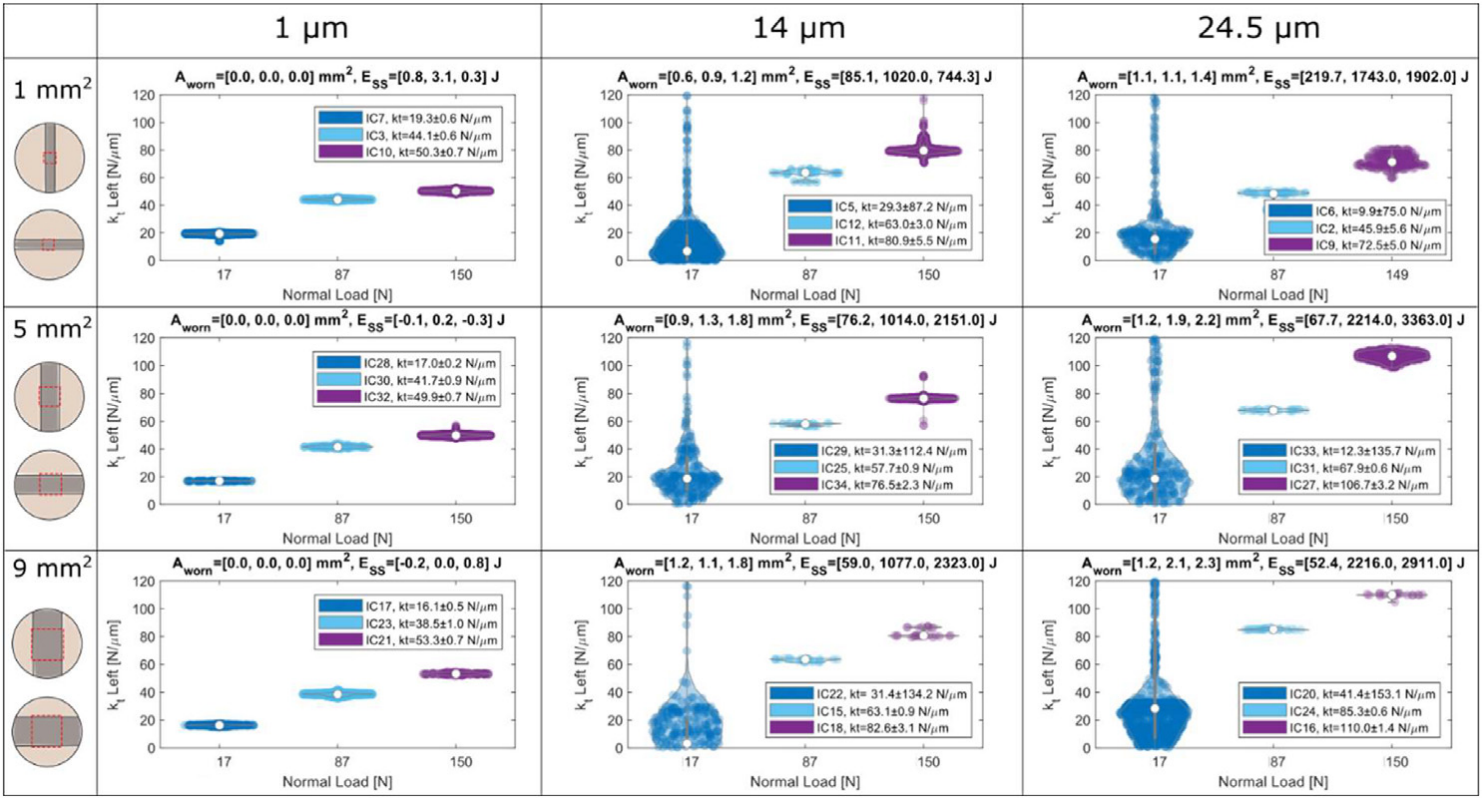
Violin plots of the steady state values of the tangential contact stiffness of every test on the Imperial friction rig. Each violin corresponds to a different test for one normal load, displacement amplitude and nominal area of contact. The legends indicate the mean and standard deviation of the steady state values of each test. The plot titles indicate the worn areas of the three tests and the energy dissipated when the steady state was reached (note that in most of the tests the steady state was not reached, and the energy represents the energy towards the end of the test). Chattering is often observed during tests at 17 N normal load and large displacement amplitudes, and that is why the standard deviation is quite large in those tests.

*Script “plot_SUMMARY_violin_figs.m”:* This script plots the steady state values of friction coefficient and tangential contact stiffness for all tests by means of violin plots. This script has been used to plot the summary figures in the Appendix B in [1]. An example is given in Fig. 7 for the tangential contact stiffness. The input test grid of this plot should not be changed by the user, since all tests are already plotted in a grid of three columns with respectively the displacement amplitudes 1, 14 and 24.5 µm, and three rows with the nominal areas of contact 1, 5, and 10 mm2 respectively. Within each subplot, the steady state values of three different tests are represented by means of violin plots plotted against the normal load of each of the three tests.

**Folder “Politecnico di Torino”:** The PoliTO folder has the same structure as that of Imperial, i.e. with the three folders “raw_data”, “Post_processed_hysteresis” and “Matlab scripts”, and the power point “PoliTO_SUMMARY.pptx”. There are only the following differences as compared to Imperial:

• The naming of the subfolders of PoliTO tests is “1″, “2″ etc., and not “IC1", “IC2" etc.
• The raw data from PoliTO experiments is not stored in two .dat file types (*File A* and *File B* like in Imperial) but it is stored in a single file type, for each recorded batch, with a .tdms format. The file name format is as follow: “AF01_10mm2_14mum_175Hz_87N_36,000.tdms”. This naming corresponds to the following structure *TestNumber_NominalArea_Displacement_ExcitationFrequency_NormalLoad_CycleNumber*. Where:
  ○ *TestNumber* is the number of the test (they all have “AF” in front because of a previous convention);
  ○ *NominalArea* is the size of the nominal area of contact of the specimen pair (10 mm^2^ in this example);
  ○ *Displacement* is the displacement amplitude of the hysteresis loops (14 µm in this example). The displacement amplitude is defined in Fig. 2;
  ○ *ExcitationFrequency* is the excitation frequency from the shaker (175 Hz in PoliTO tests);
  ○ *NormalLoad* is the normal load applied to the contact (87 N in this example);
  ○ *CycleNumber* is the number of the recorded cycle (36000^th^ hysteresis cycle in this example, which means roughly 3 min of running time for a 175 Hz excitation).
  For each tested specimen pair there are hundreds of .tdms files and each .tdms file contains the time signals of several consecutive hysteresis loops (see Fig. 1) and also other test information such as the loading conditions. These files can be read with the MATLAB^Ⓡ^ script “postprocess_raw_data_tdms.m” which is saved in the “Matlab scripts” folder. The script uses the functions in the folder “Politecnico di Torino\Matlab scripts\functions\Read_TDMA” to extract data from the .tdms files. Among the recorded test data saved within the .tdms files, the most important are the friction force and the tangential relative displacement vectors, which are needed to plot the hysteresis loops. The functioning of the “postprocess_raw_data_tdms.m” script is described in the section “Post processing with MATLAB^Ⓡ^ scripts”, and it is similar to the “postprocess_raw_data_fileA.m” used for the Imperial raw data. This script loads all the .tdms files for a given tested specimen pair and post-processes the hysteresis loops to automatically extract the contact parameters (friction coefficient and tangential contact stiffness) as already explained for the Imperial tests. For the PoliTO tests, there is no “postprocess_raw_data_fileB.m” either.
• Each hysteresis loop is made of 571 datapoints (due to 175 Hz excitation frequency and 100 kHz sampling frequency), while in Imperial each hysteresis loop was made of 600 datapoints (due to 100 Hz excitation frequency and 60 kHz sampling frequency). Therefore, matrix and vectors usually have one dimension with 571 datapoints rather than 600.
• The power point named “PoliTO_SUMMARY.pptx” in the “Politecnico di Torino” folder is structured in the same way as the “Imperial_SUMMARY.pptx”, but with one more analysis on the worn area of contact, which is presented in the last slides of the presentation.
• The rest of files and folders are the same as already described for the Imperial tests.

## 4 Experimental Design, Materials and Methods

**Fig. 8:**
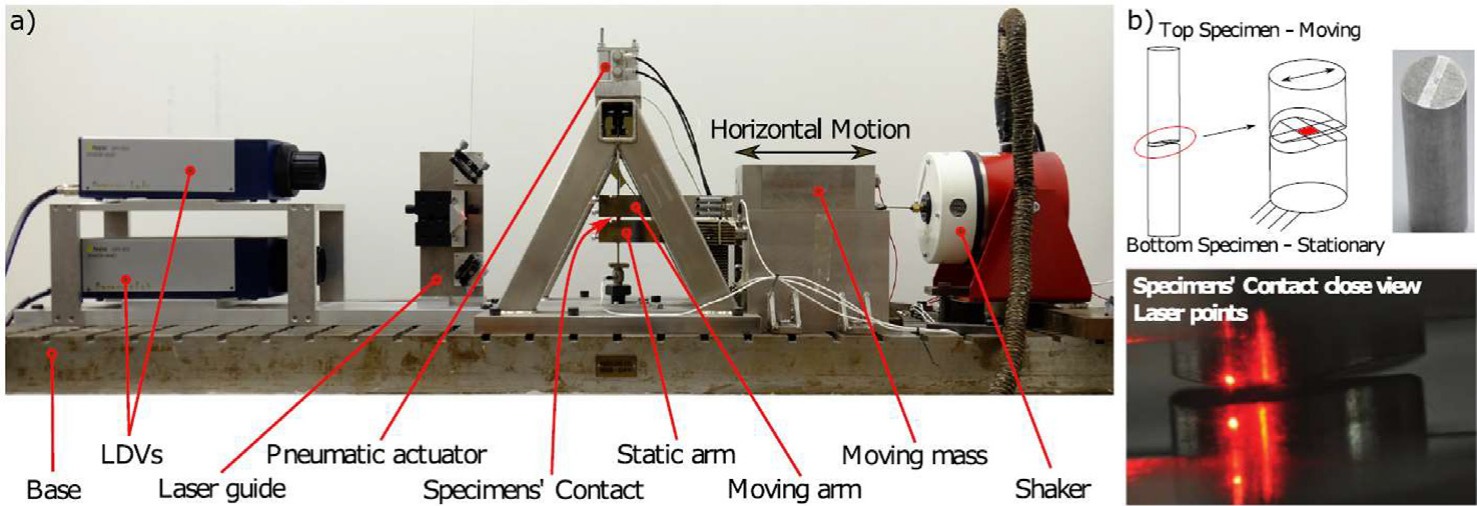
a) Imperial friction rig [3]; b) Imperial specimens: one-patch square contact.

**Fig. 9:**
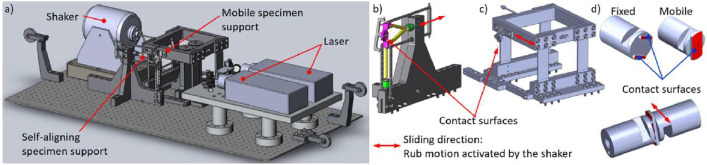
PoliTO friction rig [4]: a) overall view; b) floating self-aligning specimen support; c) mobile specimen support excited by the shaker; d) PoliTO specimens: contact occurs on the two legs.

In this study, hysteresis loops were measured from the two different fretting rigs of Imperial College London (Imperial) [3] and Politecnico di Torino (PoliTO) [4], which are shown in Fig. 8 and Fig. 9 respectively. Although their setup is quite different, the general idea behind the measurement is similar. Both rigs are excited harmonically with a shaker that generates an oscillating sliding motion between two contacting specimens. In the Imperial rig, one specimen is clamped to a moving block (see moving mass and moving arm in Fig. 8). As the reciprocating motion begins, the specimen rubs over another specimen clamped to a static block (see static arm in Fig. 8). The moving block is connected to the ground by means of two very flexible leaf springs that enable large horizontal displacements when the block is excited by the shaker. Also in the PoliTO rig, one specimen is clamped to a moving block connected to the ground by means of leaf springs (see mobile specimen support in Fig. 9). However, the second specimen is not clamped to a static block, but to a more flexible holder that enables a contact self-alignment of the specimen interfaces (see self-alignment specimen support in Fig. 9). In both rigs, the relative displacement between the specimens is measured with Laser Doppler Vibrometers (LDVs) pointing very close to the contact interfaces to minimise the bulk deformation compliance. The high accuracy of the LDVs enables to measure the relative displacement between the two specimens with up to 0.05 µm accuracy. The tangential friction force transmitted at the contact is measured with dynamic load cells that connect the specimen holders to the ground. Specimens are held in contact by applying a normal load, with a pneumatic actuator in the Imperial rig and with dead weights in the PoliTO rig. Table 1 summarises the operating regimes of the rigs.

**Table 1:** Operating regimes of Imperial and PoliTO friction rigs.

	Imperial	PoliTO
Operating frequency	100 Hz	175 Hz
Displacement amplitude pk-pk at the operating frequency	0.5–25 µm	0.5–50 µm
Nominal contact area	1–25 mm^2^	5–50 mm^2^
Nominal contact pressure	Up to 500 MPa	Up to 50 MPa

**Fig. 10:**
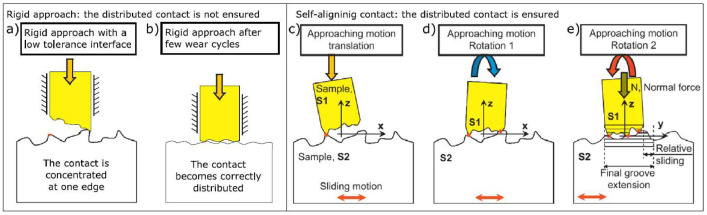
a-b) Rigid body contact approach of Imperial rig; c-e) Self-aligning contact approach of PoliTO rig.
• Contact geometry: one-leg contact vs two-leg contact. Because of the self-aligning system, PoliTO specimens employ a two-leg contact interface, as shown in Fig. 9d to ensure stability, while the Imperial rig employs a simpler one-patch square contact interface, as shown in Fig. 8b. Both contacts are nominally flat in the presented round robin test.

In addition to the slightly different operating ranges, the rigs present two main design differences:

• Contact approach: rigid alignment vs self-alignment. The Imperial rig employs a classic rigid body contact approach, in which one mobile specimen moves rigidly towards a restrained specimen, only along the pre-defined normal direction in this rig. By using this technique, the area of contact strongly relies on the tolerance of the specimen contact interfaces, which, if not perfectly parallel to each other, will most likely give a point/line contact as shown in Fig. 10a. To transform the initial point contact into a flat distributed contact, several hysteresis cycles are required until the point contact extends to surface contact due to wear (see Fig. 10b). This approach leads to easy mounting and assembly, but it requires high-tolerance interfaces to guarantee a fully distributed flat contact, at least at the beginning of the test. If high-tolerance interfaces are not achievable, a certain running-in is required to establish a distributed contact. In contrast, the PoliTO rig uses a self-alignment system for the specimens, which avoids the possibility of point or line contacts. Self-alignment is achieved by introducing two additional degrees of freedom (rotations) as shown in Fig. 10c-e. In this case, the specimens are self-adjusting to each other as long as contact occurs on at least three points that define the contact plane (a detailed description of this mechanism can be found in [4]). A disadvantage of this contact approach is that the mounting procedure is more challenging and time-consuming than the procedure of a rigid approach. By nature, this system also provides more mounting flexibility, which can make an accurate stiffness measurement more challenging.

**Fig. 11:**
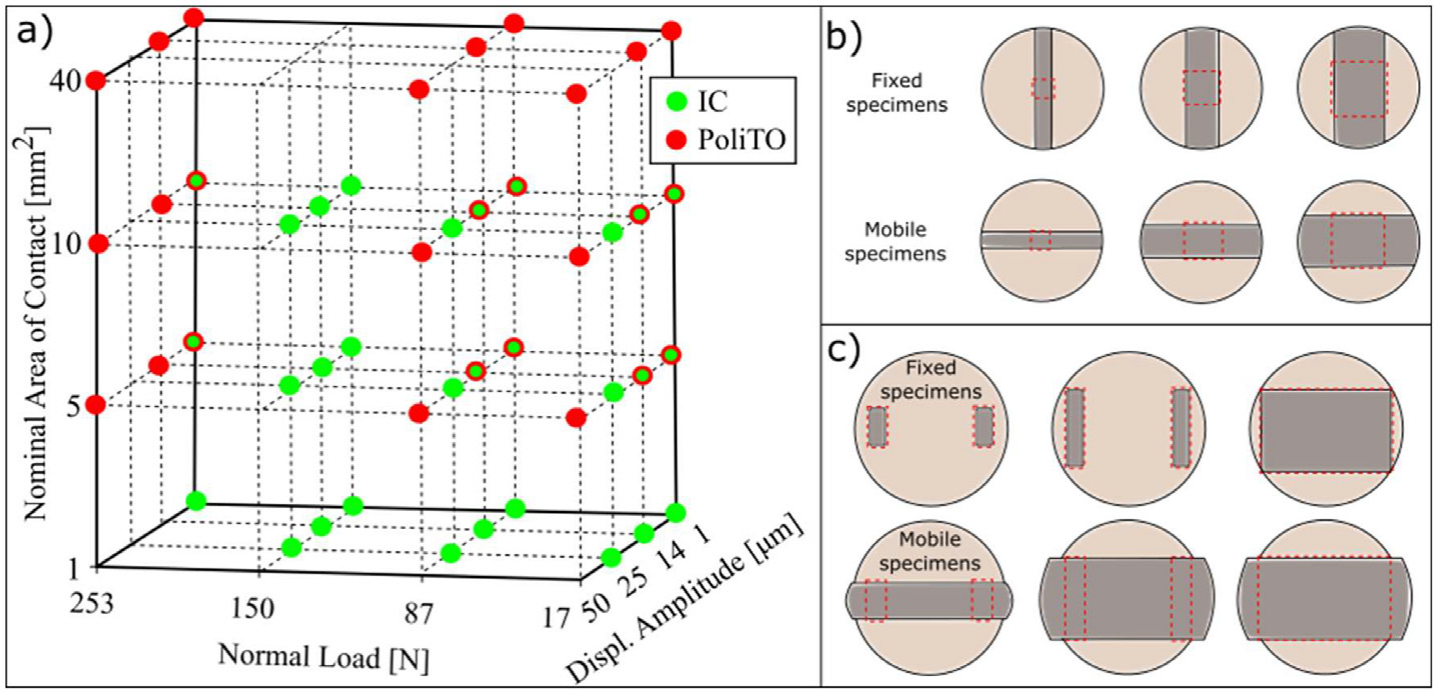
a) Test Matrix: tests with both colours were performed on both rigs; b) Specimens of Imperial, from left to right 1 mm^2^, 5 mm^2^ and 10 mm^2^; c) Specimens of PoliTO, from left to right 5 mm^2^, 10 mm^2^ and 40 mm^2^.

A large test matrix was designed to record hysteresis loops at room temperature for a wide range of test conditions. The test matrix is shown in Fig. 11a, and consisted of four normal loads (17, 87, 150 and 253 N), four displacement amplitudes pk-pk (1, 14, 25 and 50 µm) and four nominal areas of contact (1, 5, 10 and 40 mm^2^). In order to explore the widest possible experimental space and provide the largest set of data, each rig was tested at the extreme loading conditions that it could achieve. As a result, there was an overlap for 10 test conditions (circles with both green and red colours in Fig. 11a). The ranges of normal loads and displacement amplitudes were chosen to measure hysteresis loops in all the different contact regimes, namely full stuck, microslip and gross slip. The two rigs operate at slightly different excitation frequencies (100 Hz and 175 Hz), which correspond to the optimal excitation frequencies of each rig. A preliminary analysis was performed to investigate the effect of the excitation frequency on each rig. It highlighted that results were heavily dependent on the individual dynamic response of the rigs and hence it was decided to test at the optimal excitation frequencies only. Those excitation frequencies resulted in different peak sliding velocities of the specimens, obtained by multiplying the excitation frequency in [rad/s] by the pk-pk displacement amplitude divided by 2. To allow a comparison with similar sliding velocities, two of the displacement amplitudes were selected to provide the same peak velocity of 7.70 mm/s, resulting from 24.5 µm at 100 Hz for Imperial and 14 µm at 175 Hz for PoliTO, as shown in Table 2. In the designed test matrix, the lowest achievable velocity was 0.3 mm/s, which corresponds to the velocity of a stuck contact under a 100 Hz excitation.

**Table 2:** Test matrix summary.

	Units	Imperial	PoliTO
Material	–	Stainless steel 304	Stainless steel 304
Type of contact	–	Flat-on-flat (one leg)	Flat-on-flat (two legs)
Type of contact approach	–	Rigid body	Self-alignment
Temperature	–	Room temp.	Room temp.
Nominal areas of contact	[mm^2^]	1/5/10	5/10/40
Normal loads	[N]	17/87/150/254	17/87/254
Contact pressure range	[MPa]	1.7–254	0.4–51
Excitation frequency	[Hz]	100	175
Displacement amplitude pk-pk	[µm]	1/14/24.5	1/14/50
Peak sliding velocity	[mm/s]	0.31/4.40/7.70	0.55/7.70/27.5
Starting interface roughness	[µm]	0.5	5
Running time	[h]	2.5	2.5

For every test condition, a new unworn specimen pair was used. Specimens were manufactured from the same batch of raw 304 stainless steel to guarantee comparability of the material. Each test lasted 2.5 consecutive hours to achieve a proper running, which was previously shown to be adequate to achieve stable contact conditions [5]. This led to more than 1.5 million hysteresis loops per test. Before and after every test, scans of the contact interfaces were acquired with optical microscopes. More information about the interface topography is given in [1]. After 2.5 h of testing, the large number of wear cycles leads to a more distributed contact over the whole nominal contact interface. The extension of the wear at the interface was quantified through the Digital Surf Mountains^Ⓡ^ software, by selecting the black worn spots and evaluating their extension.

### 4.1 Data post-processing with MATLAB^Ⓡ^ scripts

MATLAB^Ⓡ^ scripts and functions have been written to automate the extraction of friction coefficient and tangential contact stiffness, for increased robustness and efficiency. They are stored in the “Maltab scripts” folders for both Imperial and PoliTO. In the Imperial folder, the code is called “postprocess_raw_data_fileA.m” and in the PoliTO folder is called “postprocess_raw_data_tdms.m”. They are only different in their first section where they read respectively .dat and .tdms file formats, since the two different rigs saved recorded data with those different formats.

**Fig. 12:**
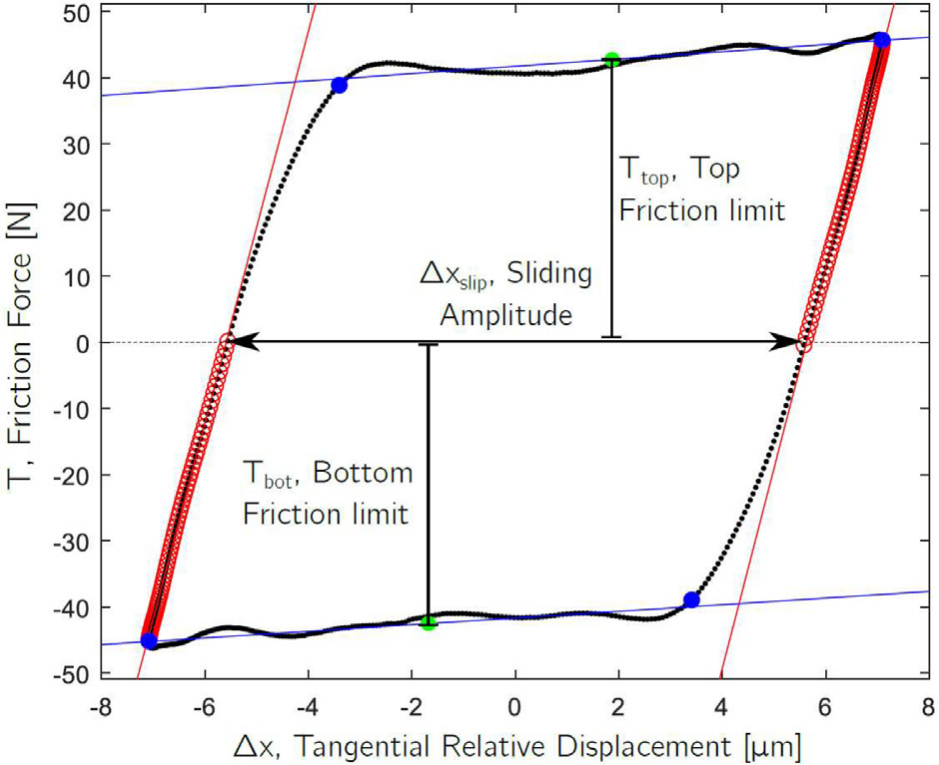
Fitting lines used for the extraction of the contact parameters. Two red lines fit the stick part of the loop and two blue lines fit the friction limits.

Both scripts load all the batches of recorded hysteresis loops for a given tested specimen pair, and then post-process the hysteresis loops to automatically extract the friction coefficient and tangential contact stiffness. The Imperial script integrates the tangential relative velocity of the specimen pairs to obtain their relative displacement. In the case of PoliTO, the relative displacement is already saved in the recorded raw data. Then, both scripts post-process all the hysteresis loops and automatically extract friction coefficient and tangential contact stiffness. The contact parameter extraction is performed with the custom MATLAB^Ⓡ^ function “func_extract_contact_parameters.m” in the folder “Matlab scripts\functions” for both rigs. The function works by fitting four lines to the hysteresis loop: two red lines that fit the stick part of the loop and two blue lines that fit the friction limits, as shown in Fig. 12. From those lines, the contact parameters are extracted as follows:

• The tangential contact stiffness, *k_t_*, is calculated as the gradient of the stick portion of the loop from the point of motion reversal to the point where the force is equal to zero. Fig. 12 shows, with red circles, the points used for the linear fitting in the stick region of the hysteresis loop. The value of the slope of the fitted red line is the *k_t_* for both left and right stick regions.
• The friction coefficient, *µ*, is calculated with two methods, the energy method and the standard method. Both methods are used here since some studies in the literature use only the first method and others only the second [6]. In this way, results could be compared to more studies. The methods are described as follows:
  ○ Energy loss formula [4], *µ=E/(2NΔx_slip_)*, where *E* is the energy dissipated within the hysteresis loop (equal to the area inside the loop, i.e. the integral of the friction force over the relative displacement), *N* is the normal load and *Δx_slip_* is the sliding amplitude, evaluated between the two points with zero friction force as shown in Fig. 12.
  ○ Standard friction coefficient with the equation *µ=(T_top_+T_bot_)/2* *N*, where *T_top_* and *T_bot_* are the (absolute) average top and bottom friction limits as shown in Fig. 12.
• In addition to *k_t_* and *µ*, the dissipated energy for each loop is evaluated as the area inside the hysteresis loop, i.e. the integral of the friction force over the relative displacement. The cumulative dissipated energy is obtained by summing up the energy dissipated by each loop during a test. Since not all loops were recorded, it was assumed that the missing hysteresis loops dissipated the same energy as the first available loop recorded after them. The cumulative energy enables a comparison of the results obtained under different test conditions (e.g. 1 hour of testing performed at 24.5 µm displacement amplitude would lead to more dissipated energy, and hence more wear, than a 1-hour test performed at 1 µm of displacement amplitude).

Since the contact parameter extraction can be a source of uncertainty in addition to the uncertainty coming from the experiments, a sensitivity study has been performed and described in the Appendix A in [1], which showed that the post-processing technique to extract contact parameters leads to a much lower uncertainty than the inherent experimental uncertainty.

**Fig. 13:**
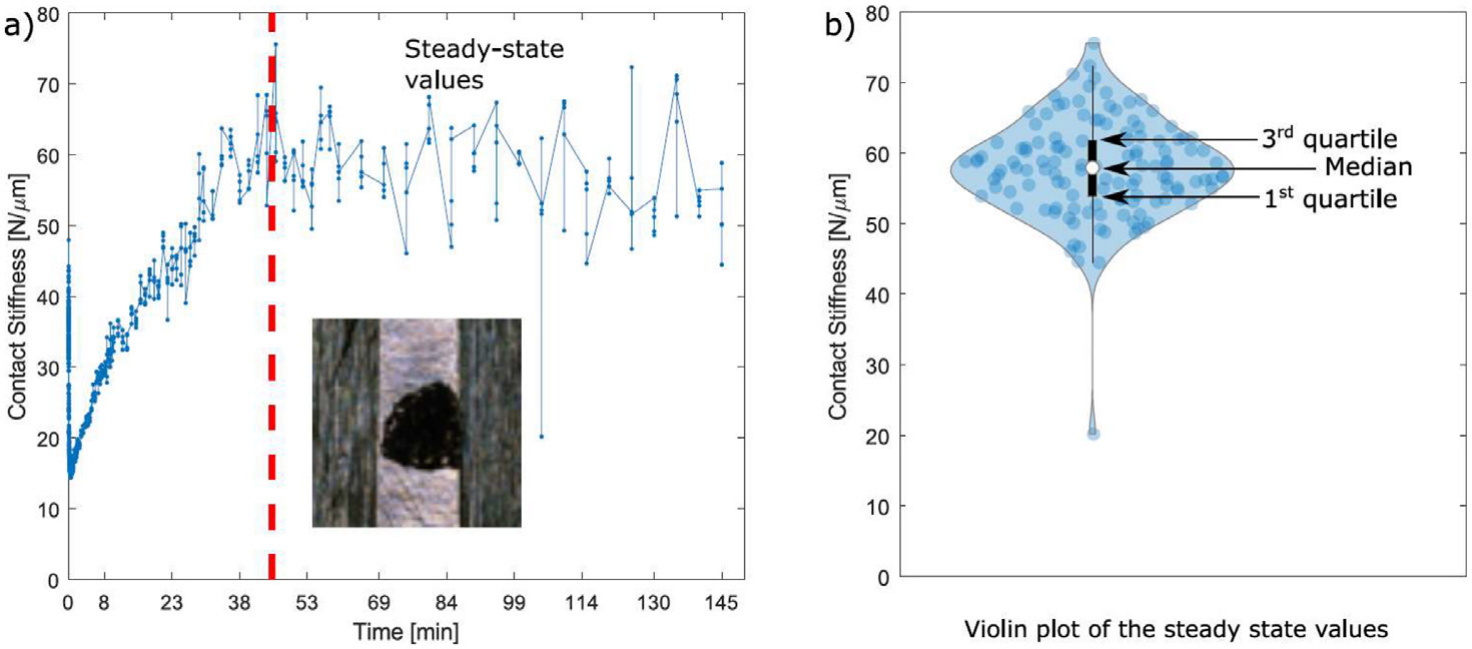
a) Typical evolution of the contact stiffness during a 2.5 h long test, and identification of the steady state values. The specimen interface at the end of the test is also shown; b) Violin plot of the steady state values of the test in a). The white marker indicates the median of the data; the black marker indicates the interquartile range (between the 1^st^ and 3^rd^ quartiles); the blue shaded area includes all sample points.

Once friction coefficient and tangential contact stiffness values have been obtained for every hysteresis loop of the analyzed test, the code saves them in the folder “Post_processed_hysteresis,” described in the previous sections. It is possible to read that post-processed data with the code “get_steadystate_values.m,” identical for both Imperial and PoliTO, which plots the evolution of the contact parameters during the test. An example of a typical trend is shown in Fig. 13a. The code asks the user to manually mark the beginning of the steady state (as indicated by the vertical red dashed line) and then a statistical analysis is performed on the steady state values after the red line. Violin plots [7] were used to plot the statistical distribution of the steady state values since they provide an understanding of the data distribution, while not taking more space than box plots. As an example, Fig. 13b shows the violin plot of the steady state values of the test in Fig. 13a. As an example, violin plots of the steady state values of the tangential contact stiffness for every test on the Imperial friction rig were shown in Fig. 7 and in the summary figures of the Appendix B in [1].

## Limitations

Tests presented here were performed on stainless steel specimens at room temperature under the loading conditions defined in Table 2. More work is required in future to expand the test range of the presented round robin and generalise the findings. In addition, some tests presented the following issues during raw data recording (documented in the log files in each individual test folder in the “raw_data” folders for both Imperial and PoliTO tests):

• Loss of laser focus due to wear debris or technical failure. This led to non-reliable measurements. This data was excluded from the analysis.
• Large amount of noise due to chattering during excitation. This led to post-processing issues since the contact parameters could not be extracted.

**Fig. 14:**
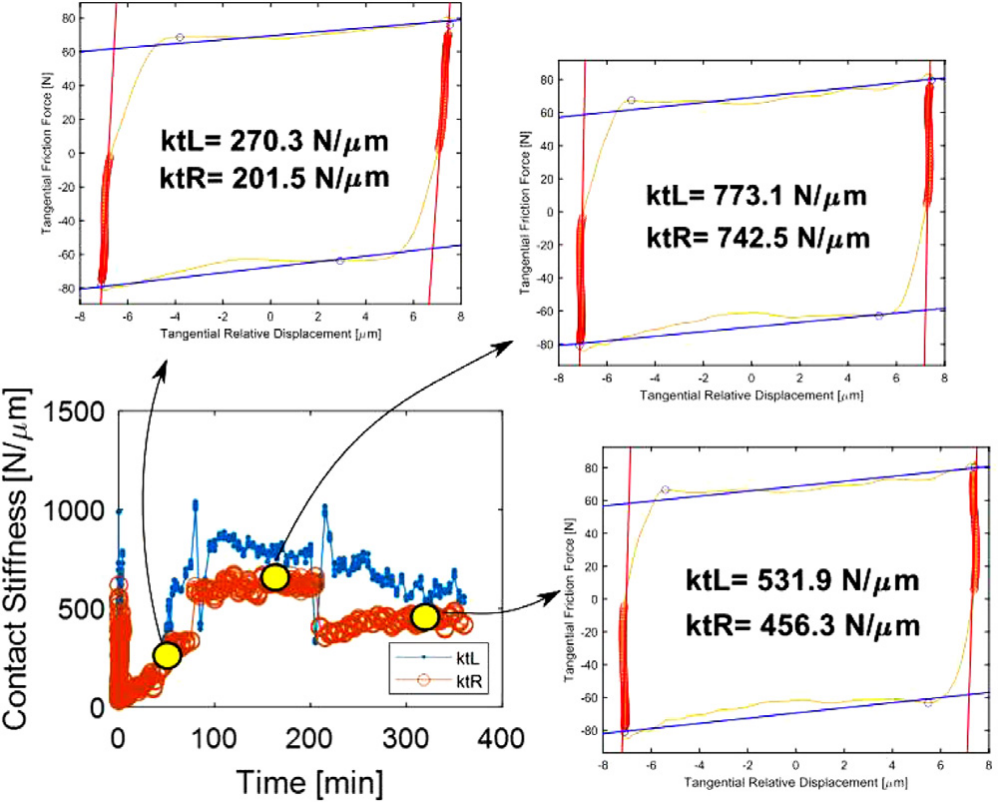
Example of post-processing issues in the kt estimations occurring in few PoliTO tests with very large kt (>300 N/µm). Test n. 5: 87 N normal load, 14 µm displacement amplitude, 10 mm^2^ nominal area of contact, 175 Hz excitation frequency and 6 h of running.

Post-processing issues: some of the PoliTO hysteresis loops had very large contact stiffnesses, above 300 N/µm. In those cases, even minimal changes in the measured displacement led to major changes in the *k_t_* estimated from the force-displacement slope. For example, Fig. 14 shows that, even though the loops look similar through the test, slight changes in the measured displacement, due to noise, lead to massive changes in the estimated stiffness. Some of those loops are shown in the “PoliTO_SUMMARY.pptx”, see e.g. slides 40 and 41. The reasons for these relatively high measured values have been discussed in the related research article [1], e.g. in sections 5 and 6.

## Ethics Statement

The authors have read and follow the ethical requirements for publication in Data in Brief and confirm that the current work does not involve human subjects, animal experiments, or any data collected from social media platforms.

## CRediT authorship contribution statement

**Alfredo Fantetti:** Conceptualization, Methodology, Software, Investigation, Writing – original draft. **Daniele Botto:** Conceptualization, Funding acquisition, Resources, Supervision, Writing – review & editing. **Christoph Schwingshackl:** Conceptualization, Funding acquisition, Resources, Supervision, Writing – review & editing. **Stefano Zucca:** Conceptualization, Funding acquisition, Resources, Supervision, Writing – review & editing.

## Data Availability

Dataset of contact parameters and hysteresis loops from a round robin test for nonlinear dynamic analysis (Original data) (Mendeley Data).
